# Anterior mitral leaflet perforation: a rare complication of radiofrequency ablation for paroxysmal supraventricular tachycardia

**DOI:** 10.1002/ccr3.1037

**Published:** 2017-06-30

**Authors:** Jiancheng Han, Jin Xu, Yihua He

**Affiliations:** ^1^ Department of Ultrasound Beijing Anzhen Hospital Capital Medical University Beijing China

**Keywords:** Anterior mitral leaflet, perforation, radiofrequency ablation

## Abstract

A 9‐year‐old girl with a ten‐day history of palpitations was referred for the assessment of mitral regurgitation. She had had RF ablation for paroxysmal supraventricular tachycardia 5 years previously. Echocardiography showed isolated anterior mitral leaflet perforation. Surgical findings confirmed the echocardiographic assessment.

A 9‐year‐old girl with a ten‐day history of palpitations was referred for the assessment of mitral regurgitation. She had had RF ablation for paroxysmal supraventricular tachycardia (PSVT,left accessary pathway) 5 years previously. Her cardiac auscultation revealed a III/VI systolic murmur at the left upper sternal border with no other abnormalities. Two‐dimensional transthoracic echocardiography and color flow mapping showed isolated anterior mitral leaflet (AML) perforation (Fig. 1A) with severe mitral regurgitation (MR) (Fig. 1B). Real‐time three‐dimensional transthoracic echocardiography viewed from the left ventricle (LV) revealed a hole in the A2 region near the anterior annulus of the AML (Fig. 2A). Real‐time three‐dimensional color flow full volume imaging showed severe MR originating from the AML perforation (Fig. 2B). Surgical findings confirmed the echocardiographic assessment. The perforation was repaired with a patch of fresh autologous pericardium. Postoperative echocardiography showed trace central mitral regurgitation.

**Figure 1:**
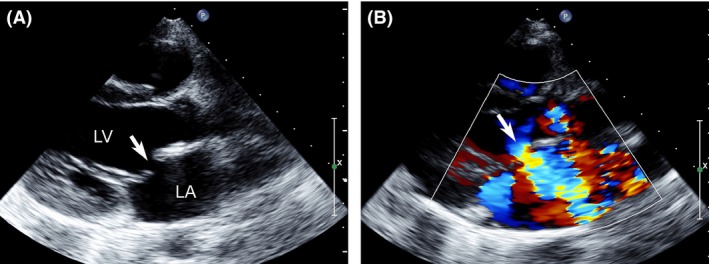
Parasternal left ventricular long‐axis view: Two‐dimensional transthoracic echocardiography showed anterior mitral leaflet perforation (arrow). Parasternal left ventricular long‐axis view: Two‐dimensional color Doppler imaging showed severe mitral regurgitation (arrow). LA, left atrium; LV, left ventricle.

**Figure 2:**
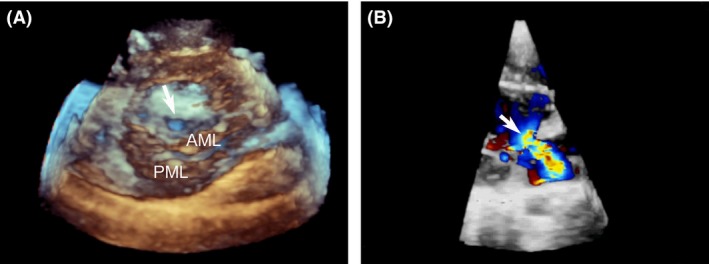
Real‐time three‐dimensional transthoracic echocardiography revealed a hole in the A2 region (arrow) near to the anterior annulus of the anterior mitral leaflet (AML). Real‐time three‐dimensional color flow full volume imaging showed severe MR originated from anterior mitral leaflet perforation (arrow). PML, posterior mitral leaflet.

## Authorship

JH: performed the echo and wrote the manuscript. JX: collected the clinical data. YH: provided the scientific direction of the manuscript.

## Conflict of Interest

None declared.

